# Chronic central nervous system aspergillosis with coexisting GFAP and AQP4 antibody positivity: case report and literature review

**DOI:** 10.3389/fimmu.2025.1613142

**Published:** 2025-07-04

**Authors:** Yimin Cao, Xueying Zhao, Shengpu Hao, Haiqing Yang, Duo Gao, Shixuan Du, Liang Wang, LiXia Zhou

**Affiliations:** ^1^ Department of Medical Imaging, The Second Hospital of Hebei Medical University, Shijiazhuang, Hebei, China; ^2^ Department of Pathology, The Second Hospital of Hebei Medical University, Shijiazhuang, Hebei, China; ^3^ Department of Neurology, The Second Hospital of Hebei Medical University, Shijiazhuang, Hebei, China

**Keywords:** autoimmune antibodies, central nervous system aspergillosis, immunity, aquaporin-4 antibody, glial fibrillary acidic protein antibody

## Abstract

This article presents an unusual case of chronic central nervous system (CNS) aspergillosis with concurrent glial fibrillary acidic protein (GFAP)-IgG and aquaporin-4 (AQP4)-IgG seropositivity. The patient presented with progressive numbness and weakness of the extremities over three months. The diagnosis was established by a cell-based assay demonstrating double positivity for GFAP-IgG and AQP4-IgG; imaging revealed multiple foci in the right temporo-occipital lobe and cervical medulla. Surgical pathology confirmed parenchymal *Aspergillus* infection. Postoperative treatment with voriconazole was effective. This case represents, to our knowledge, the first report of chronic CNS aspergillosis with concurrent GFAP-IgG and AQP4-IgG positivity. These findings suggest that chronic fungal infections may induce multiple distinct antibody responses, thereby offering new insights into the mechanisms linking infection and immunity. Clinical vigilance is therefore warranted for the development of autoimmune antibodies following chronic fungal infections, and an early diagnosis may be facilitated by integrating clinical, imaging, and pathological evaluations.

## Introduction

Central nervous system (CNS) aspergillosis is a rare but fatal invasive fungal infection, primarily affecting immunocompromised individuals (1). While neurological injury in CNS aspergillosis has traditionally been attributed to direct fungal invasion and vascular damage, emerging evidence suggests that infectious insults may also trigger or unmask autoimmune responses within the CNS. *Aspergillus* species can invade the brain through multiple mechanisms, including transcellular and paracellular migration. Upon entering the CNS, *Aspergillus* triggers a cascade of immune responses in which resident CNS cells—such as microglia and astrocytes—recognize fungal components via surface-expressed pattern recognition receptors (PRRs), initiating inflammatory signaling (2).

Although infection-induced autoimmunity has garnered increasing attention, the precise relationship between fungal infections and astrocyte-specific antibodies (e.g., Glial fibrillary acidic protein and Aquaporin-4 antibodies) remains poorly elucidated. AQP4 and GFAP antibodies serve as key biomarkers for neuromyelitis optica spectrum disorders (NMOSD) and autoimmune GFAP astrocytopathy, respectively—two classical astrocytopathies (3). This potential pathophysiological overlap poses significant diagnostic challenges, as severe infections may be misdiagnosed as primary autoimmune diseases, thereby leading to inappropriate immunosuppressive therapy. Herein, we report an exceptionally rare case of CNS aspergillosis with concurrent detection of GFAP and AQP4 antibodies in cerebrospinal fluid (CSF). This case clearly illustrates that CNS fungal infections can mimic the clinical and serological features of autoimmune astrocytopathies. By highlighting this diagnostic pitfall, we aim to emphasize the complex interplay between infection and CNS autoimmunity.

The study was conducted in compliance with the Declaration of Helsinki, and ethical approval was obtained from the ethics Committee of hospital (approval number: 2025-P015). Written informed consent was obtained from the patient, which included authorization for the publication of clinical data, laboratory results, and anonymized imaging findings. All personal identifiers have been omitted or de-identified to protect patient confidentiality.

## Description of case

A 64-year-old woman presented with progressive numbness and weakness in her extremities evolving over three months, prompting hospital admission. Manual muscle testing revealed Grade 4+ strength in the proximal muscles of both upper extremities and Grade 3+ strength in both lower extremities. Neurological findings included hypoesthesia to pinprick and vibration below C4, with absent vibratory sense below C7. The biceps, triceps, brachioradialis, and abdominal reflexes were absent. Babinski’s sign was positive bilaterally. Her medical history included lumbar discectomy performed 25 years earlier complicated by histopathologically confirmed intracranial fungal infection (biopsy-proven). Although original surgical records were unavailable due to temporal constraints, The patient reported that after self-discharge, they failed to obtain medications from external pharmacies, and the symptoms subsequently alleviated spontaneously, resulting in no follow-up treatment being received. Comorbidities comprised hypothyroidism (10 years) and type 2 diabetes mellitus (5-year duration).

**Figure 1:**
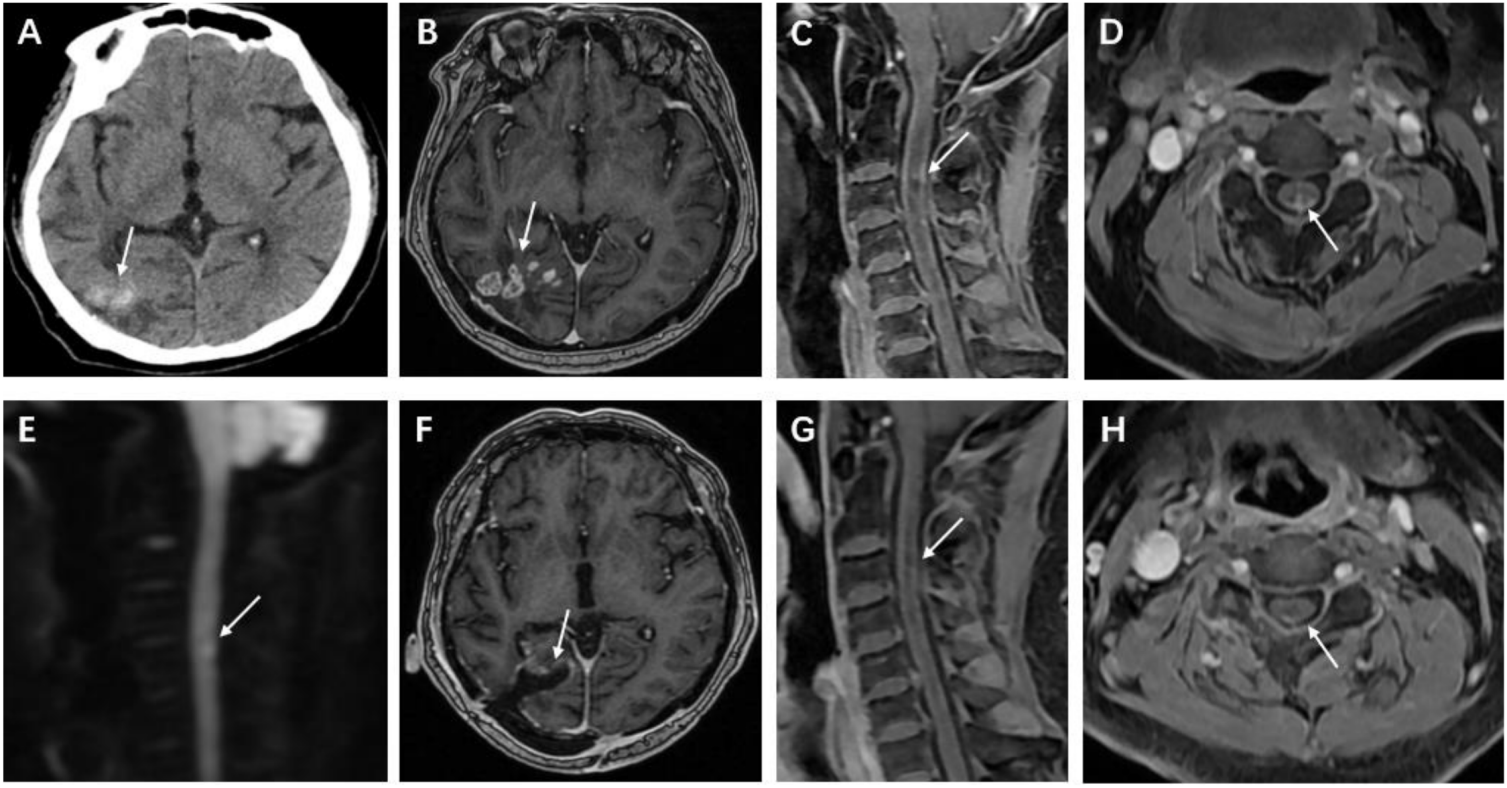
Cranial CT and MRI after hospital admission. **(A, B)** Cranial computed tomography (CT) shows a right temporoparietal-occipital lobe lesion exhibiting mixed high and low densities; contrast-enhanced examination reveals multiple circularly enhancing foci within the lesion and adjacent meningeal enhancement. **(C, D)** Spinal magnetic resonance imaging (MRI) reveals nodular enhancing foci within the spinal cord at the C3-C5 levels, accompanied by adjacent pial enhancement. **(E)** Diffusion-weighted imaging (DWI) demonstrates restricted diffusion within the spinal lesions. Follow-up brain and cervical spine MRI 8 weeks after surgery. **(F)** Postoperative MRI of the brain demonstrates changes in the right temporo-occipital lobe, with no abnormal enhancing lesions. **(G, H)** Cervical spine MRI shows that the extent of abnormal enhancing lesions in the spinal cord is reduced.

The post-admission cranial computed tomography (CT) demonstrated multiple calcified nodules within the brain parenchyma, localized to the subcortical region of the right temporo-parieto-occipital junction (**Figure 1A**). Subsequent magnetic resonance imaging (MRI) of the brain revealed multiple ring-enhancing lesions with contiguous leptomeningeal enhancement. Spinal MRI showed segmental cord expansion accompanied by intramedullary T2-weighted imaging (T2WI) hyperintensity displaying heterogeneous signal characteristics. Post-contrast sequences demonstrated nodular enhancing foci within the spinal cord parenchyma and associated pial surface enhancement. Diffusion-weighted imaging (DWI) revealed restricted diffusion within the affected spinal cord segments (**Figures 1B–E**).

**Figure 2:**
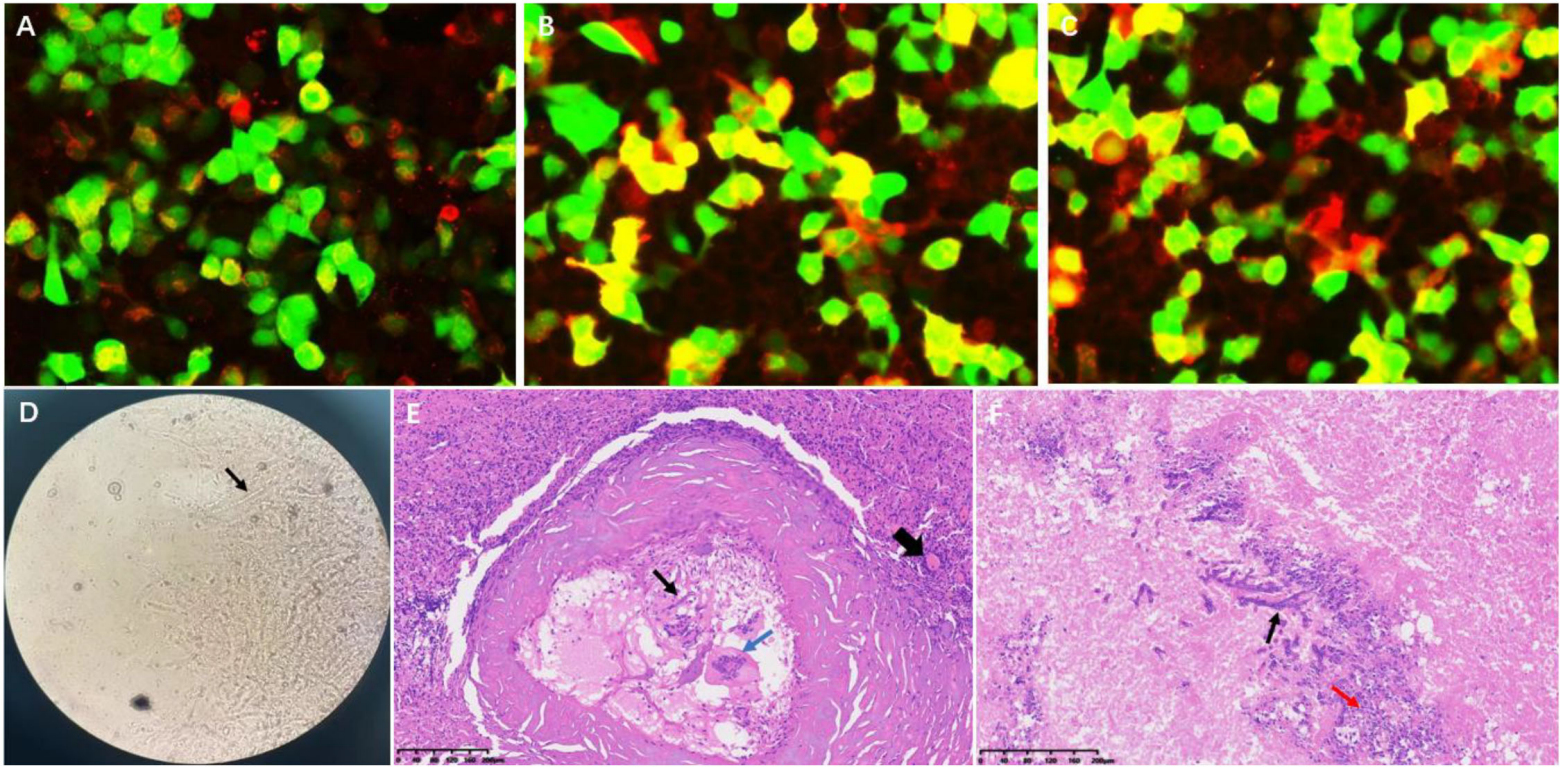
Immunological and histopathological findings. **(A)** CBA demonstrates GFAP-IgG positivity in cerebrospinal fluid (titer 1:32) (Red fluorescence). **(B, C)** Dual AQP4-IgG seropositivity in CSF and serum (titer 1:100) (Red fluorescence). **(D)** Grocott’s methenamine silver staining reveals Aspergillus hyphae (black arrow). **(E, F)** Hematoxylin-eosin staining shows: fungal hyphae (thin black arrow), spores and necrosis (red arrow), multinucleated giant cells (blue arrow), with perilesional inflammatory infiltration (dotted circle) and reactive vascular proliferation (thick black arrow).

Upon admission, laboratory tests revealed a neutrophil percentage of 75.04% (reference range: 40–75%) and a lymphocyte percentage of 16.37% (reference range: 20–40%). Serum creatinine was 162.0 μmol/L, indicating renal insufficiency. Immune function analysis showed that the total T-lymphocyte count (0.80 × 10^9^/L), CD4^+^ T-cell count (0.40 × 10^9^/L), and NK-cell count (0.075 × 10^9^/L) were all below the normal reference ranges, suggesting cellular immunodeficiency. For differential diagnosis, a series of etiological screenings were performed. Serologically, both the (1→3)-β-D-glucan assay (G test) and galactomannan assay (GM test) yielded negative results, as did the Brucella agglutination test. Cerebrospinal fluid (CSF) obtained via lumbar puncture exhibited an opening pressure of 120 mmH_2_O. Biochemical analysis of CSF demonstrated a marked elevation in protein concentration (1.16 g/L; reference range: 0.15–0.45 g/L), while tests for cryptococcal antigen, Mycobacterium tuberculosis complex, and viral DNA remained negative. GFAP-IgG, assessed by cell-based assay (CBA), was positive in the CSF at a titer of 1:32. AQP4-IgG was positive in both the CSF and serum at a titer of 1:100 (**Figures 2A–C**). CSF revealed a type IV pattern of oligoclonal bands, characterized by an absence of intrathecal synthesis, a peripheral B-cell immune response, and concomitant blood-brain barrier disruption.

A multidisciplinary consultation concluded that the brain parenchymal lesion likely represented a fungal infection, and the possibility of demyelinating lesions in the spinal cord could not be excluded. Due to the limited extent of the intramedullary lesion and the risk of severe injury associated with a spinal cord biopsy, an excisional biopsy of the right temporo-occipital lobe lesion was performed ten days after admission. Pathological examination revealed fungal hyphae and spores within the lesional brain tissue, accompanied by infiltration of numerous inflammatory cells and glial cell hyperplasia (**Figures 2D–F**). Metagenomic sequencing (DNA and RNA) of brain tissue for pathogenic microorganisms revealed *Aspergillus* genus (956 sequences detected, relative abundance: 49.95%) and *Aspergillus* fumigatus species (603 sequences detected, relative abundance: 31.5%). The final clinical diagnosis was CNS aspergillosis.

The patient received oral voriconazole (Due to renal dysfunction, oral administration was chosen.) as continuous antifungal therapy postoperatively, initiated at 400 mg twice daily during hospitalization. The dosage was titrated to 200 mg twice daily upon discharge based on therapeutic drug monitoring. At 8-week follow-up, neurological assessment revealed symptomatic amelioration with partial recovery of lower limb muscle strength, with the grade improving from 3/5 to 4/5. Follow-up MRI demonstrated complete resolution of postoperative parenchymal and leptomeningeal lesions, accompanied by significant regression of spinal cord lesions and mild pial surface enhancement (**Figures 1F–H**). Repeat laboratory evaluation showed mild leukocytosis. Cerebrospinal fluid analysis revealed normal cell counts and biochemical parameters.

## Discussion

Central nervous system (CNS) aspergillosis poses diagnostic challenges due to its overlapping clinical and radiological features with inflammatory demyelinating disorders and neoplastic conditions (4). Notably, the patient’s medical history included histologically confirmed cerebral aspergillosis 25 years prior, complicated by multiple chronic comorbidities. The patient demonstrated compromised immunity, evidenced by a total T-lymphocyte count at the lower limit of normal, a borderline-low CD4^+^ T-cell count, and a markedly reduced NK-cell count. These findings collectively indicate impairments in cellular and innate immune function, supporting a high risk of CNS aspergillosis. The definitive diagnosis of recurrent CNS aspergillosis was established through triangulation of history of Aspergillus infection, characteristic neuroradiological findings (multifocal, annularly enhancing lesions involving the meninges), and histopathological identification. Preoperative multidisciplinary consensus deemed the spinal cord lesions diagnostically equivocal, given the concurrent GFAP-IgG and AQP4-IgG positivity raising suspicion of autoimmune disease. However, three critical findings confirmed the infectious etiology: (1) intraoperative specimens demonstrated invasive Aspergillus hyphae on Grocott’s methenamine silver staining; (2) intramedullary lesions exhibited distinctive imaging manifestations; (3) post-antifungal therapeutic response manifested as complete resolution of spinal cord lesions on follow-up MRI. To our knowledge, this represents the longest-documented disease course of CNS aspergillosis with dual GFAP/AQP4 antibody positivity reported in the medical literature.

The pathogenesis of CNS aspergillosis is characterized by necrotizing granulomas and abscess formation following vascular infiltration (5). In the present case, the protracted nature of the disease was evidenced by the presence of foci of cranial calcification, granuloma formation, and meningeal involvement (6). Spinal cord infection may result in the formation of an inflammatory infiltrate, granulomas, and spinal meningeal involvement; fungal hyphae cause cellular infiltration, manifesting as diffusion restriction within the lesion on imaging. The diagnosis of spinal aspergillosis was substantiated by MRI of the spinal cord lesions in this case.

The most notable finding in this case was the concurrent seropositivity for both GFAP and AQP4 antibodies. We hypothesize that this phenomenon represents not a coincidental comorbidity of two distinct disorders, but rather a sequential immune cascade initiated by Aspergillus infection.

GFAP, the primary structural protein in astrocytes, maintains cellular mechanical strength and supports neuronal function through its intermediate filament network. The polarized distribution of GFAP within astrocytic perivascular endfeet is crucial for regulating the localization of AQP4, which is predominantly enriched at the basement membrane, thereby contributing to the maintenance of blood-brain barrier (BBB) homeostasis (7). Gliotoxin (GTX), a mycotoxin produced by *Aspergillus* species, can disrupt astrocytic perivascular endfeet, leading to a loss of BBB integrity and increased permeability. Furthermore, cerebrospinal fluid (CSF)/plasma GFAP levels in infected patients show a correlation with the extent of BBB damage (2, 8). Following BBB disruption, GFAP can enter the bloodstream; this exposure of GFAP may subsequently trigger immune responses and enhance the antigen-presenting capabilities of astrocytes (9). Similarly, the exposure of AQP4 due to BBB breakdown can trigger an elevation in AQP4-IgG levels, suggesting a synergistic interaction. Certain viral infections, such as Epstein-Barr virus (EBV), are known to induce the expression of both GFAP and AQP4 antibodies (10). However, to date, there have been no reports of concurrent positivity for both AQP4 and GFAP antibodies following *Aspergillus* infection. We hypothesize that fungal infections, potentially via a ‘GTX → BBB damage → antigen exposure → immune dysregulation’ cascade, may induce dual or multiple autoimmune responses. This process could involve the exposure of antigens (such as GFAP and AQP4) and an inflammatory microenvironment-driven immune dysregulation, collectively contributing to autoantibody production. This case report partially elucidates a potential mechanism linking *Aspergillus* infection with the production of neuroimmune autoantibodies, thereby offering novel perspectives on the interplay between neuroinflammation, infection, and autoimmunity.

The coexistence of spinal cord lesions and GFAP/AQP4 antibody seropositivity in this case necessitated critical differentiation from primary autoimmune astrocytopathies and neuromyelitis optica spectrum disorders (NMOSD).

GFAP-A: Characteristic imaging features include longitudinally extensive spinal cord lesions demonstrating multifocal intramedullary spotty or patchy enhancement on contrast imaging, often accompanied by linear leptomeningeal and nerve root enhancement. This case exhibited similar spinal MRI findings: patchy enhancing lesions and meningeal enhancement (11). NMOSD: Spinal cord lesions in NMOSD typically manifest as longitudinally extensive transverse myelitis with lesions predominantly involving central cord parenchyma, showing patchy or ring enhancement during acute phases. Despite the patient’s AQP4 antibody seropositivity, brain imaging lacked typical NMOSD features (e.g., periventricular, periependymal, thalamic, or brainstem lesions) (12).

The spinal lesion extent, diffusion restriction, combined with multiple intracranial calcifications and ring-enhancing lesions in this case were highly suggestive of infectious granulomas. Moreover, the effective response to antifungal therapy fundamentally differed from autoimmune encephalopathies requiring immunosuppressive treatment.

The patient tested negative for both G test and GM test assays. Although these assays demonstrate considerable diagnostic value for invasive aspergillosis, their sensitivity tends to decrease significantly in chronic infections. For granuloma-encapsulated CNS infections specifically, systemic antigen release may be substantially limited, thereby increasing the probability of false-negative results (13, 14). Consequently, negative serological markers in this case cannot exclude a CNS Aspergillus diagnosis, which should prompt continued pursuit of infectious etiologies when clinical suspicion remains high despite negative biomarkers. Additionally, the dynamic changes in G and GM assays still hold monitoring value during treatment, which is expected to reflect the therapeutic response and prognosis.

The therapeutic strategy in this case was established based on definitive etiological diagnosis. Given the patient’s renal insufficiency, oral voriconazole was selected for antifungal therapy. An initial loading dose of 400 mg twice daily was administered, followed by maintenance dosing adjusted to 200 mg twice daily guided by therapeutic drug monitoring (TDM). The treatment course is typically recommended for 6–12 months, requiring individualized adjustments according to clinical response, drug concentration, and imaging follow-up. At the 8-week follow-up, neurological function demonstrated mild improvement with significant radiological resolution, confirming antifungal efficacy.

A critical clinical decision involved whether to add immunosuppressants despite GFAP/AQP4 antibody positivity. Our experience indicates that when infectious etiology is confirmed and targeted antimicrobial therapy proves effective, immunosuppressants should be avoided as this may exacerbate underlying infections with potentially catastrophic consequences. This case compellingly demonstrates that prioritizing primary infection control constitutes an effective therapeutic pathway when infection coexists with autoantibodies.

## Conclusion

In summary, this study reports for the first time a histopathologically confirmed case of chronic central nervous system (CNS) aspergillosis exhibiting concurrent GFAP-IgG and AQP4-IgG seropositivity. Our findings suggest these autoantibodies likely represent an epiphenomenon triggered by chronic fungal infection rather than independent autoimmune processes. This case underscores the complexity of interpreting autoantibody profiles, particularly in the setting of active infection. It serves as a critical alert to clinicians: when evaluating patients with suspected autoimmune encephalomyelitis—especially those with atypical imaging features or predisposing risk factors—chronic occult infections (e.g., fungal etiologies) must be included in the differential diagnosis, with active pursuit of pathogenetic evidence. Future investigations should dissect the molecular mechanisms underlying fungus-induced CNS autoimmunity, potentially offering novel perspectives on the interplay between infection, immunity, and neurodegenerative disorders.

## Clinical evaluation, diagnosis, and treatment strategy

For patients suspected of chronic CNS aspergillosis with GFAP-IgG/AQP4-IgG positivity, the following clinical approach is recommended:

1. Evaluation and Differential Diagnosis

Clinical screening: Prioritize immunocompromised status (e.g., diabetes, hypothyroidism), history of fungal infection, and progressive neurological deficits.

Multimodal diagnostics:

Imaging: Brain CT/MRI for multifocal calcified nodules and ring-enhancing lesions; spinal MRI to differentiate long-segment myelitis from demyelinating diseases.

Laboratory tests: CSF analysis (protein, cell count, GFAP/AQP4-IgG) and *Aspergillus* markers (GM/G tests).

Pathogenesis confirmation: Brain biopsy (fungal hyphae in tissue) remains essential; add metagenomic sequencing for pathogen detection.

2. Treatment Protocol

Antifungal therapy (normal renal function): Voriconazole (6 mg/kg IV q12h or 400 mg PO q12h loading for 24h), then 4 mg/kg IV q12h or 200 mg PO q12h adjusted by therapeutic drug monitoring targeting trough concentrations of 2.0–5.5 μg/mL, administered for 6–12 months.

Antifungal therapy (renal dysfunction): Voriconazole (oral use only: 400 mg PO q12h loading for 24h, for adults ≥40 kg), then 200 mg PO q12h adjusted by therapeutic drug monitoring targeting trough concentrations of 2.0–5.5 μg/mL, administered for 6–12 months.

Follow-up: Serial MRI + neurological function assessments.

3. Key Insights

Coexisting autoantibodies and infectious imaging findings require pathogen evidence to avoid misdiagnosis as autoimmune diseases. Multidisciplinary consultation is essential for integrated decision-making.

## Data Availability

The original contributions presented in the study are included in the article/Supplementary Material. Further inquiries can be directed to the corresponding authors.
